# Data on the transcriptional regulation of DNA damage induced apoptosis suppressor (DDIAS) by ERK5/MEF2B pathway in lung cancer cells

**DOI:** 10.1016/j.dib.2016.08.066

**Published:** 2016-09-06

**Authors:** Joo-Young Im, Sung-Hoon Yoon, Bo-Kyung Kim, Hyun Seung Ban, Kyoung-Jae Won, Kyung-Sook Chung, Kyeong Eun Jung, Misun Won

**Affiliations:** a Personalized Genomic Medicine Research Center, KRIBB, Daejeon 305-806, Korea; b Functional Genomics, University of Science and Technology, Daejeon 305-701, Korea; c Biomedical Translational Research Center, KRIBB, Daejeon 305-806, Korea; d ST Pharm. Co., LTD, Sihwa Industrial Complex 1, Kyunggido 429-848, Korea

**Keywords:** DDIAS, ERK5, MEF2B, Lung cancer

## Abstract

The data included in this article are associated with the article entitled “DNA-damage-induced apoptosis suppressor (DDIAS) is upregulated via ERK5/MEF2B signaling and promotes β-catenin-mediated invasion” (J.Y. Im, S.H. Yoon, B.K. Kim, H.S. Ban, K.J. Won, K.S. Chung, K.E. Jung, M. Won) [1]. Quantitative RT-PCR data revealed that genetic or pharmacological inhibition of extracellular signal-regulated kinase 5 (ERK5) suppresses DDIAS transcription in response to epidermal growth factor (EGF) in Hela cells. p300 did not interact with myocyte enhancer factor 2B (MEF2B), a downstream target of ERK5 and affect transcription of DDIAS. Moreover, DDIAS transcription is activated by ERK5/MEF2B signaling on EGF exposure in the non-small cell lung cancer cells (NSCLC) NCI-H1703 and NCI-H1299. DDIAS knockdown suppresses lung cancer cell invasion by decreasing β-catenin protein level on EGF exposure.

Specifications Table

**Table:** 

Subject area	Biology
More specific subject area	Cell biology, Molecular biology
Type of data	Image, graph, figure
How data was acquired	Quantitative PCR, Western blot, Transwell invasion assay
Data format	Raw
Experimental factors	Cells were overexpressed with ERK5, DDIAS, β-catenin or transfected with siRNA against ERK1, ERK2, ERK5, MEF2B, DDIAS
Experimental features	Samples were HeLa, non-small cell lung cancer cells, NCI-H1299, NCI-H1703 cells
Data source location	Daejeon, South Korea
Data accessibility	Data is available with the article

**Value of the data**

• Transcription of DDIAS is activated by ERK5/MEF2B pathway in lung cancer cells.
• Increase of DDIAS transcription activates β-catenin signaling to promote lung cancer cell invasion.
• The data provide evidence that DDIAS is a potential therapeutic target of lung cancer.

## 1 Data

**Fig. 1:**
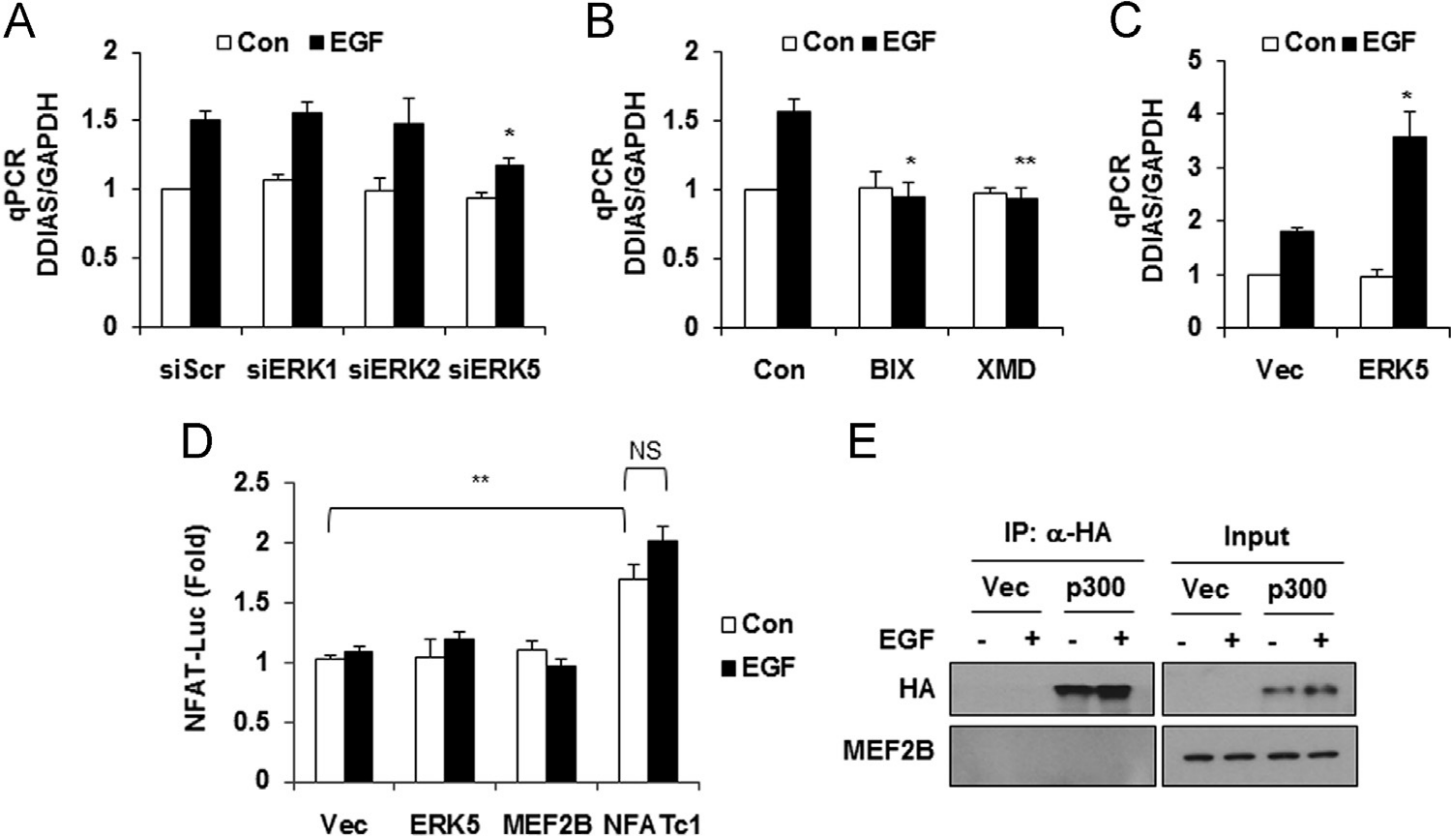
**Determination of DDIAS mRNA expression using real-time PCR in HeLa cells.** (A) ERK5 knockdown inhibited DDIAS mRNA expression. (B) MEK5 (BIX02189) or ERK5 (XMD8-92) inhibitors suppressed EGF-induced DDIAS mRNA expression. (C) Overexpression of HA-ERK5 increased EGF-induced DDIAS mRNA expression. Quantitative reverse transcription-PCR (qRT-PCR) was performed. *P<0.05, **P<0.01 vs. siScr/EGF, Con/EGF or Vec/EGF. (D) Overexpression of HA-ERK5 or Myc-MEF2B has no effect on EGF-induced NFAT promoter activity. Cells were transfected with HA-ERK5, Myc-MEF2B or HA-NFATc1 and NFAT-luciferase reporter construct (NFAT-Luc) together. **P< 0.01 vs Vec/Con. NS, no significance. (E) p300 does not interact with MEF2B. HeLa cells were transfected with HA-p300 and treated with 100 ng/ml EGF for 3 h. Immunoprecipitation assays were performed using anti-HA and detected using an anti-MEF2B antibody.

**Fig. 2:**
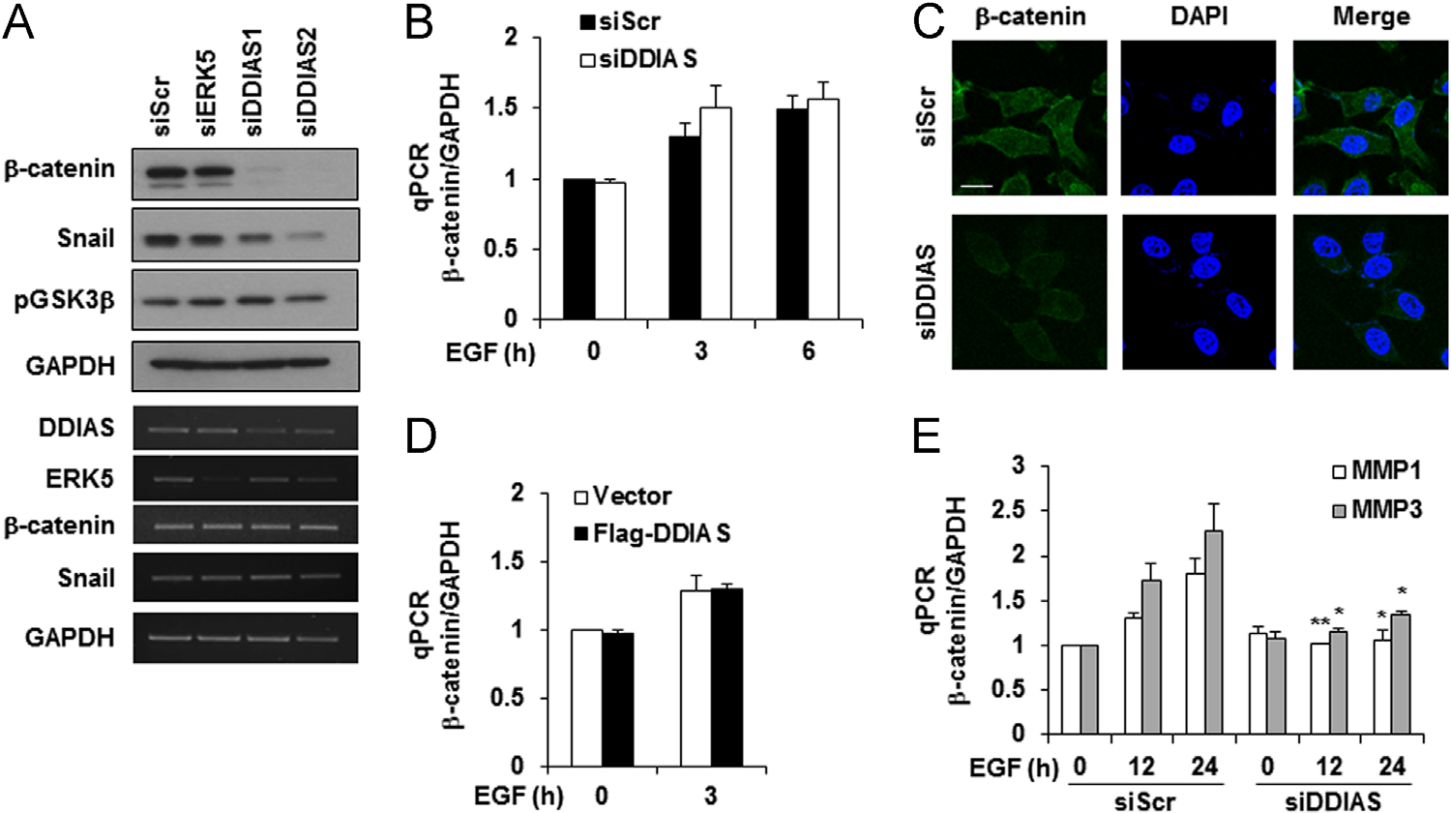
**DDIAS knockdown destabilizes β-catenin protein expression.** (A) β-catenin mRNA and protein expression. HeLa cells were transfected with siRNA against ERK5, DDIAS1 or DDIAS2 for 60 h. qRT-PCR and Western blotting analyses were performed. (B) The effect of DDIAS on β-catenin mRNA expression. (C) β-catenin staining in DDIAS knockdown cells. HeLa cells were transfected with 20 nM of DDIAS siRNA for 60 h. Immunocytochemistry was performed using an anti-β-catenin antibody. The cell nuclei were stained with DAPI. Scale bar, 20 μm. (D) The effect of Flag-DDIAS overexpression on β-catenin expression. (E) MMP1 or MMP3 expression following DDIAS knockdown. HeLa cells were treated with EGF for the indicated times, and qRT-PCR was performed. The values represent the mean±SEM of three independent experiments. *P<0.05, **P< 0.01 vs. siScr/EGF.

**Fig. 3:**
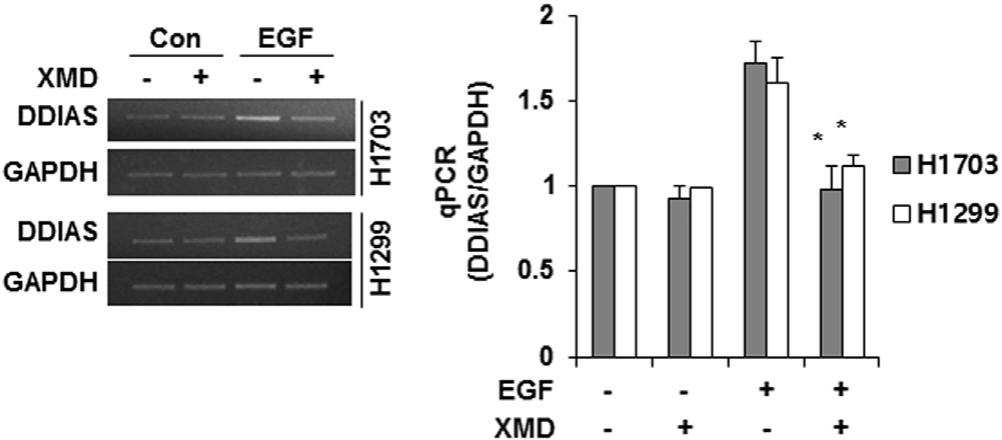
**Inhibition of ERK5 suppresses DDIAS expression in lung cancer cells**. NCI-H1703 (H1703) and NCI-H1299 (H1299) cells were pretreated with XMD8-92 for 1 h and then incubated with 100 ng/ml of EGF for 12 h. qRT-PCR was performed. The values represent the mean±SEM of three independent experiments performed in triplicate. *P<0.05 vs. EGF.

**Fig. 4:**
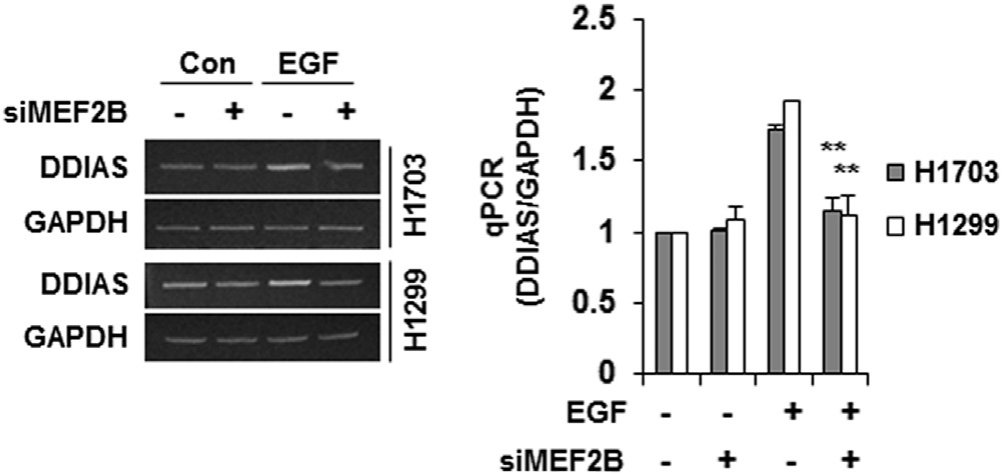
**MEF2B knockdown suppresses EGF-induced DDIAS expression in lung cancer cells.** H1703 and H1299 cells were transfected with 40 nM of siScr or siMEF2B for 48 h and then incubated with 100 ng/ml of EGF for 12 h. qRT-PCR was performed. The values represent the mean±SEM of three independent experiments performed in triplicate. *P<0.05 vs. EGF.

**Fig. 5:**
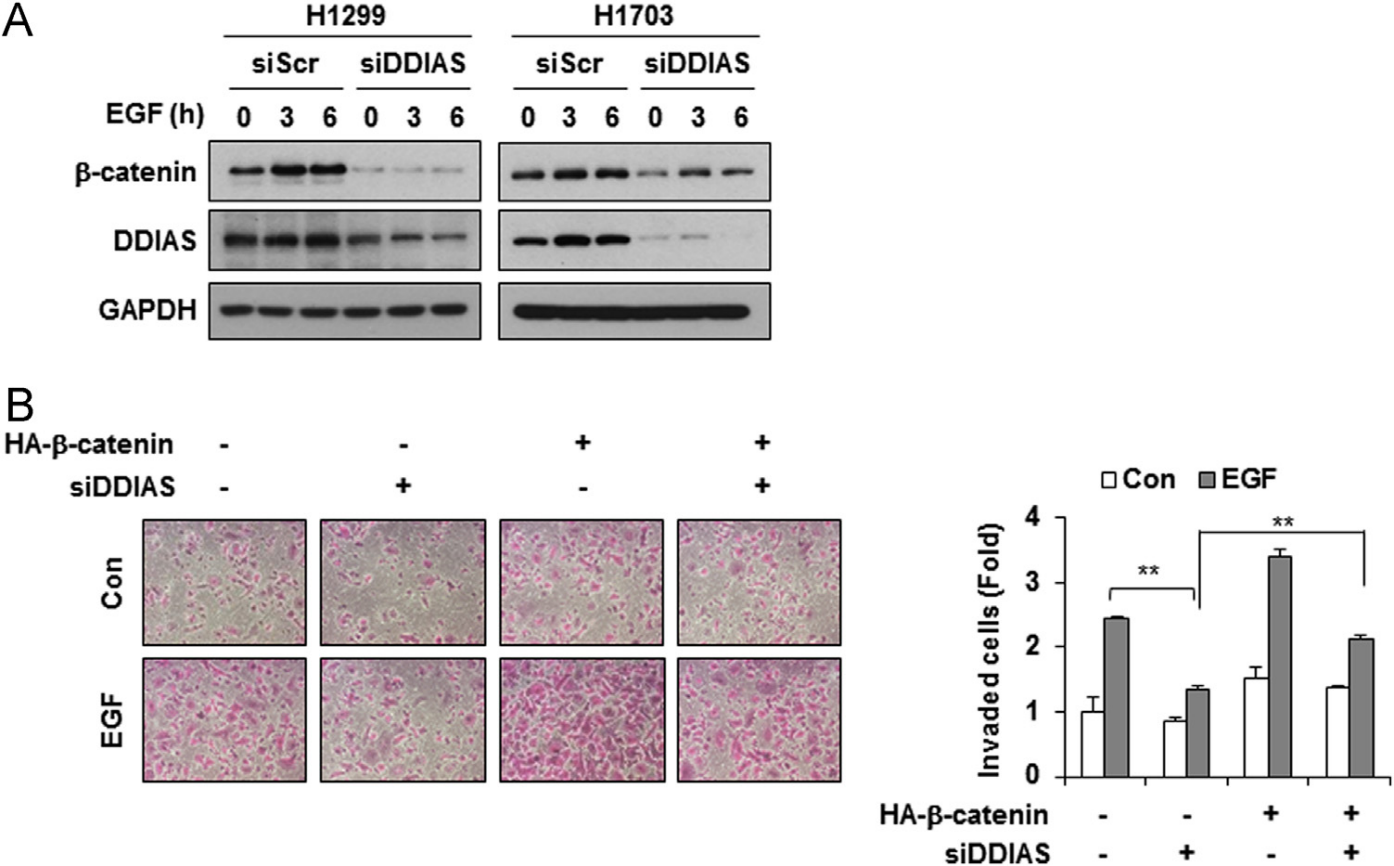
β**-catenin protein expression following DDIAS knockdown**. (A) DDIAS knockdown suppresses EGF-induced β-catenin protein accumulation in NCI-H1703 and NCI-H1299 cells. Western blotting was performed using anti-β-catenin, anti-DDIAS or anti-GAPDH antibodies. (B) β-catenin overexpression restores the cell invasion repressed by DDIAS knockdown. NCI-H1299 cells were transfected with 20 nM of siScr or siDDIAS for 24 h and then transfected with vector control or HA-β-catenin for 24 h. The values represent the mean±SEM of two independent experiments performed in triplicate. **P< 0.01 vs. siScr/EGF.

DDIAS is highly expressed in lung cancers and is involved in cisplatin resistance [2], [3]. In HeLa cells, genetic and pharmacological inhibition of MEK/ERK5 suppressed EGF-induced DDIAS transcription, whereas ERK5 overexpression increased DDIAS mRNA level (Fig. 1). DDIAS knockdown dramatically decreased β-catenin protein level in HeLa cells (Fig. 2). Consistent with data in HeLa cells, inhibition of ERK5 suppressed DDIAS transcription on EGF exposure in lung cancer cell lines (Fig. 3). In addition, MEF2B knockdown reduced EGF-induced DDIAS expression in lung cancer cells (Fig. 4). Furthermore, DDIAS knockdown inhibited β-catenin accumulation and lung cancer cell invasion (Fig. 5).

## 2 Experimental design, materials and methods

### 2.1 Cell culture and transfection

HeLa cells were cultured in Dulbecco׳s modified Eagle׳s medium and non-small cell lung cancer cell, NCI-H1703 and NCI-H1299 cells were cultured in RPMI-1640 containing 10% fetal bovine serum (FBS), 50 U/mL of penicillin, and 50 μg/mL of streptomycin (Invitrogen, Carlsbad, CA, USA) in an incubator at 37 °C and 5% CO_2_. Knockdown and overexpression of target genes experiment were performed as described [1]. Cells were transiently transfected with HA-ERK5, HA-p300, Flag-DDIAS or HA-β-catenin using Turbofect (ThermoScientific, Rockford, IL) [4].

### 2.2 RT-PCR

Total RNA extraction and Real-time PCR were performed as described [1]. The cycling conditions were 95 °C for 15 min and 40 cycles of 95 °C for 15 s, 55 °C for 15 s and 72 °C for 15 s. All reactions were performed in triplicate and normalized to GAPDH as an internal control. The values are presented as the mean±S.E.M.

### 2.3 Co-immunoprecipitation assays

Co-immunoprecipitation assay was performed as previously described [5]. Cell lysates were immunoprecipitated with agarose-conjugated anti-HA antibody. Then, Western blot analyses were carried out using antibodies to peroxidase-conjugated anti-HA or anti-MEF2B antibodies.

### 2.4 Immunocytochemistry

Immunocytochemistry analysis was performed as described [1]. The fixed cells were incubated with anti-β-catenin antibody in 1% BSA solutions. Cells were then incubated with fluorescein-conjugated secondary antibodies (FITC, Santa Cruz). Finally, the cells were counterstained with DAPI to label nuclei and were then analyzed with a fluorescence microscope (LSM5 Live DuoScan, Zeiss).

### 2.5 Invasion assay

Invasion assay was performed as described [1]. Serum-starved cells (1×10^5^) were seeded in a Matrigel-coated chamber (BD Biosciences, Palo Alto, CA, USA) with 8.0-μm pores (Corning) with or without 100 ng/ml EGF. More than five views were analyzed under a light microscope. All experiments were performed twice in triplicate.
